# Dietary Flavonoids: Cardioprotective Potential with Antioxidant Effects and Their Pharmacokinetic, Toxicological and Therapeutic Concerns

**DOI:** 10.3390/molecules26134021

**Published:** 2021-06-30

**Authors:** Johra Khan, Prashanta Kumar Deb, Somi Priya, Karla Damián Medina, Rajlakshmi Devi, Sanjay G. Walode, Mithun Rudrapal

**Affiliations:** 1 Department of Medical Laboratory Sciences, College of Applied Medical Sciences, Majmaah University, Al Majmaah 11952, Saudi Arabia; j.khan@mu.edu.sa; 2 Life Sciences Division, Institute of Advanced Study in Science and Technology, Guwahati 781035, Assam, India; shaandeb2010@gmail.com (P.K.D.); rajlakshmi@iasst.gov.in (R.D.); 3 Department of Pharmaceutical Sciences & Technology, Birla Institute of Technology, Mesra, Ranchi 835215, Jharkhand, India; 4 University Institute of Pharmaceutical Sciences, Panjab University, Chandigarh 160014, India; somipriya99@gmail.com; 5 Food Technology Unit, Centre for Research and Assistance in Technology and Design of Jalisco State A.C., Camino Arenero 1227, El Bajío del Arenal, Zapopan 45019, Jalisco, Mexico; karla.damian03@gmail.com; 6 Rasiklal M. Dhariwal Institute of Pharmaceutical Education & Research, Chinchwad, Pune 411019, Maharashtra, India; sanjuwalode@rediffmail.com

**Keywords:** dietary flavonoids, cardioprotective effects, ROS scavenging, myocardial dysfunction, bioavailability and drug metabolism, toxicity, drug discovery

## Abstract

Flavonoids comprise a large group of structurally diverse polyphenolic compounds of plant origin and are abundantly found in human diet such as fruits, vegetables, grains, tea, dairy products, red wine, etc. Major classes of flavonoids include flavonols, flavones, flavanones, flavanols, anthocyanidins, isoflavones, and chalcones. Owing to their potential health benefits and medicinal significance, flavonoids are now considered as an indispensable component in a variety of medicinal, pharmaceutical, nutraceutical, and cosmetic preparations. Moreover, flavonoids play a significant role in preventing cardiovascular diseases (CVDs), which could be mainly due to their antioxidant, antiatherogenic, and antithrombotic effects. Epidemiological and in vitro/in vivo evidence of antioxidant effects supports the cardioprotective function of dietary flavonoids. Further, the inhibition of LDL oxidation and platelet aggregation following regular consumption of food containing flavonoids and moderate consumption of red wine might protect against atherosclerosis and thrombosis. One study suggests that daily intake of 100 mg of flavonoids through the diet may reduce the risk of developing morbidity and mortality due to coronary heart disease (CHD) by approximately 10%. This review summarizes dietary flavonoids with their sources and potential health implications in CVDs including various redox-active cardioprotective (molecular) mechanisms with antioxidant effects. Pharmacokinetic (oral bioavailability, drug metabolism), toxicological, and therapeutic aspects of dietary flavonoids are also addressed herein with future directions for the discovery and development of useful drug candidates/therapeutic molecules.

## 1. Introduction

Cardiovascular diseases (CVDs) are the most prominent cause of death across the world. Over three-quarters of deaths due to CVDs take place in low- and middle-income countries. An estimated 17.9 million people died from CVDs in 2016, constituting 31% of all global deaths. Of these deaths, 85% are due to heart attack and stroke [1]. Most of the CVDs can be prevented by tackling behavioral risk factors such as tobacco use, insalubrious diet and obesity, physical inactivity, and long consumption of alcohol using population-wide approaches. In the United States, for example, lack of awareness towards leading a healthy lifestyle contributes to nearly half of all cardiometabolic disorders [2]. In India, premature mortality because of CVDs has increased from 37 million in 2010 to 52 million in 2020. In Western populations, only 23% of CVD deaths occur before the age of 70 years while in India this number is 52% [3]. The World Health Organization (WHO) estimation demonstrates that over 75% of premature CVD is preventable, and that associated risk factors betterment can help decrease the mounting CVD burden on both people and healthcare workers [4]. Autopsy evidence suggests that the progression of CVDs in later years is not foreseeable, and thus management is crucial. The INTERHEART study explicated the consequences of CVD risk factors including dyslipidemia, hypertension, diabetes, abdominal obesity, smoking, at the same time as it demonstrated the shielding effects of consumption of nutritious fruits and vegetables, and regular exercise. People with cardiovascular disease or who are at high cardiovascular risk, including those having an already-established disease like hypertension, diabetes, hyperlipidemia, etc., require early recognition and management using appropriate counseling and medications [5].

Diet and lifestyle have an eminent effect on LDL-cholesterol levels and CVD risk. Patients with CVDs should be counseled about lifestyle modifications to reduce fat and cholesterol ingestion, to avoid tobacco products, and to maintain the caloric level in their body by ensuring appropriate physical activity in order to maintain a healthy BMI. A body mass index (BMI) > 25 is a risk factor for CVDs, with the lowest probability at BMI 20–25, while a BMI < 20 is not routinely recommended [6]. In the prevailing years, it has been reported that the majority of cardiovascular diseases occur due to an imbalance between the formation of reactive oxygen species (ROS) and ROS-degrading antioxidant systems. This disparity results in the accrual of superoxide, hydrogen peroxide, and other by-products such as peroxynitrite and hypochlorous acid, which leads to oxidative damage of vital cell structures and essential biomolecules including lipids, membranes, proteins, and DNA. This phenomenon causes deactivation of essential metabolic enzymes and also destroys signal transduction pathways [7]. Oxidative stress (OS) has been linked to a variety of diseases, including neurodegenerative disorders, autoimmune diseases, complex lifestyle diseases, and cancer, and it is implicated in the pathogenesis of over 100 inflammatory disorders, including diabetes, rheumatoid arthritis, periodontitis, stroke, CVDs, and alveolar inflammations. In general, there are numerous molecular mechanisms involving sources of ROS and their respective targets. One intracellular site for ROS generation is the mitochondrial electron transport chain where the generation of ROS takes place due to the leakage of a small fraction of electrons to oxygen. Antioxidants present in the mitochondria including superoxide dismutase (SOD) and glutathione sequester ROS to reduce their reactivity [8]. Cardiac tissues hold a large number of mitochondria, but the antioxidant capacity is not sufficient enough for sequestering ROS, which results in cardiac dysfunction or mitochondrial cellular oxidative stress. It has been proven that oxidized low-density lipoprotein (ox-LDL) increases the development of ROS [9] in human umbilical vein endothelial cells (HUVECs). Angiotensin II and uremic toxin indoxylsulfate-induced endothelial cell dysfunction are two other recognized causes of ROS noticed in CVDs [10].

It has been well established from previous reports that sugars are involved in the development of atherosclerosis, hypertension, peripheral vascular disease, coronary artery disease, cardiomyopathy, heart failure, and cardiac arrhythmias, and that these effects of added sugars are mediated through ROS, as glucose can produce ROS via various pathways including the sorbitol pathway, insulin pathway, and NADPH-oxidase (Nox) pathway. Nox signaling is crucial for normal physiology, but overstimulated Nox enzymes contribute to oxidative stress and cardiovascular disease [11]. In AT-II-induced hypertension, NOX-2 activation induces sirtuin-3 (SIRT3) S-glutathionylation which causes acetylation of vascular SOD2 and reduces SOD2 activity, which further results in increased mitochondrial superoxide levels and lessened endothelial nitric oxide bioavailability which acts as an antioxidant in vivo [11,12].

A diet low in saturated fat and high in fruits, vegetables, and essential fatty acids, as well as moderate wine intake, appears to protect against the production and progression of CVDs, according to epidemiological evidence [13]. Long-term metabolic studies have shown that the fatty acid composition of the diet, rather than the overall amount of fat consumed, predicts serum cholesterol levels. Saturated fatty acids (SFA) and trans fatty acids are the ones associated with elevated cardiovascular risk; however, monounsaturated fatty acids (MUFA, omega-9) and polyunsaturated fatty acids (PUFA, omega-3, omega-6) explicitly decreased the risk of coronary heart disease (CHD) [13]. The activity of enzymes involved in the desaturation of fatty acids in the body is highly influenced by dietary fat quality. Plant sterols and stanols (saturated form of sterols) are natural elements of plants structurally related to cholesterol. Plant stanols lessen cholesterol absorption in the GIT thereby dipping plasma LDL concentrations. These stanols are found abundantly in vegetable oils, olive oil, fruits, and nuts. Recent progressions in food technology have perceived the emergence of nutrition products such as margarine, milk, yoghurt, and cereal products being supplemented with plant sterols/stanols and being encouraged as a food that can help lower serum cholesterol [14]. It has been found via clinical studies that serum LDL cholesterol significantly dropped when stanols were added to milk (15.9%) and yoghurt (8.6%), but dropped significantly less when added to bread (6.5%) and cereal (5.4%). Nonetheless, routine consumption of phytosterols has emerged as an effective strategy in the management of hypercholesterolemic patients in the clinical situation. Alternatively, red yeast rice (*Monascus purpureus*) is a natural compound capable of reducing cholesterol levels. This fermented rice holds plentiful monacolins that are naturally occurring HMG-CoA reductase inhibitors [15]. The commercial preparations of this traditional supplement possess a beneficial lipid-lowering effect. Several studies including cohort studies have suggested a J-shaped relationship between salt intake and CVD risk. As per the recommendation of WHO, gradual salt reduction in one’s diet represents an attainable, cost effective, and efficient strategy to prevent CVD worldwide. The INTERSALT study (an international study of electrolyte excretion and BP) confirmed a direct association between salt intake and the increase in BP with age [16].

Despite many previous published reports on flavonoids (including dietary flavonoids) and their health benefits/biological potential in various human diseases such as cancer, neurodegenerative diseases, CVDs, etc., there are no clear reports available in current literature that indicate biochemical mechanisms of action, or the pharmacokinetic and toxicological profile, of dietary flavonoids associated with cardioprotective effects. In view of this, the aim of this paper was to review the cardioprotective effects of dietary flavonoids summarizing their antioxidant potential in OS/ROS-induced CVDs including biochemical mechanisms of action, pharmacokinetic and toxicity issues, and therapeutic/nutraceutical approaches with future directions in the discovery of drugs or therapeutic candidates.

## 2. Dietary Flavonoids

### 2.1. Dietary Occurrence

**Figure 1:**
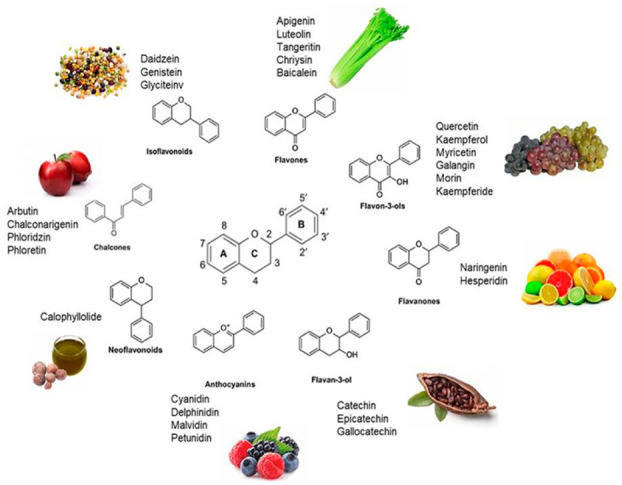
Dietary sources of flavonoids.

Flavonoids are secondary metabolites located in the vacuoles of plants. Approximately 10,000 flavonoids have been reported in the literature, positioning them in third place of the most abundant bioactive compounds in plants. The main function of flavonoids in plants is to protect plants against pathogens and UV radiation, and to participate in pollination by being recognized by pollinators [17]. Flavonoids’ basic chemical structure consists of 15 carbon atoms (C_6_-C_3_-C_6_) making up the two aromatic rings A and B linked by a C ring consisting of 3 carbon atoms (Figure 1).

The classification of flavonoids can be done according to the position of the carbon in the B ring linked with the C ring. Thus, the flavonoids linked in position 3 of the C ring are denominated isoflavones, the ones linked in position 4 are neoflavonoids, and, finally, those linked to position 2 are subdivided into different subgroups (flavones, flavonols, flavanones, flavanonols, flavanols, anthocyanins, and chalcones), depending on the structural characteristics of the C ring [18]. Flavonols, such as quercetin, kaempferol, and myricetin, are one of the most common flavonoids found in fruits and vegetables, including apples, grapes, berries, tomatoes, onions, lettuce, etc. The chemical structure of flavonols is characterized by a ketone group and a hydroxyl group located in position 3 of the C ring, which can have different glycosylation patterns. For these reasons, the flavonoid subgroups are the largest subgroups present in plants and foods [19].

On the other hand, the most well-known compounds in the flavanones group are hesperidin, naringenin, and eriodyctiol, which are regularly found in the white part of the peel of citrus fruits such as lemon, orange, and grapefruit. Structurally, these compounds are very similar to flavonols; the only difference is the saturation of the C ring in the 2 and 3 position [19].

Isoflavonoids are less distributed throughout plants, and are usually present in lentils, beans, soybean, and other leguminous plants. The most important bioactive compounds on this group are genistein and daidzein, which are well known as a phytoestrogen due to their osteogenicactivity [18].

Neoflavonoids are a less studied group. Their structure is characterized by a 4-phenylchromen backbone with no hydroxyl group substitution at position 2. The hydroxyl group is bound to position 3 of the C ring [18]. One of the neoflavones is calophyllolide from *Calophyllum inophyllum* seeds, found in other plants and flowers [20]. Flavanols like catechins are abundantly distributed in berries, bananas, peaches, and apples.

**Figure 2:**
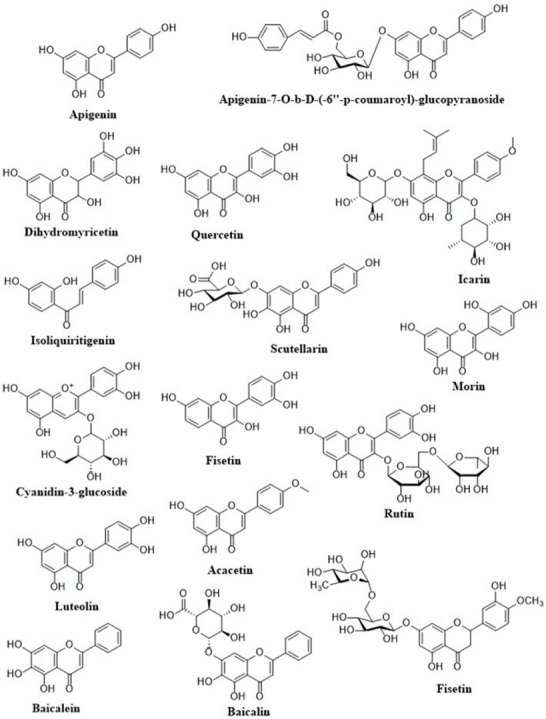
Structures of dietary flavonoids.

**Figure 3:**
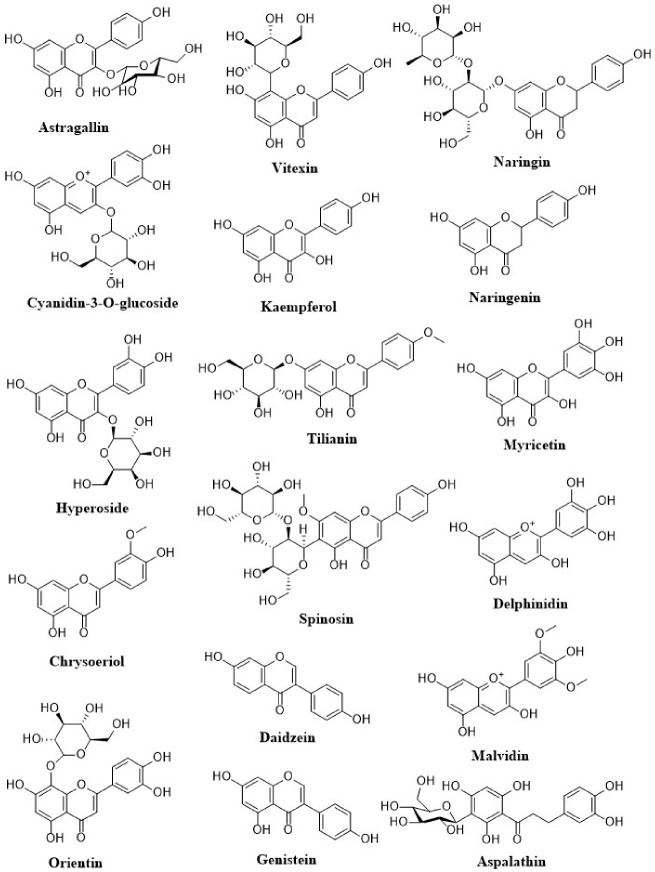
Structures of dietary flavonoids.

**Figure 4:**
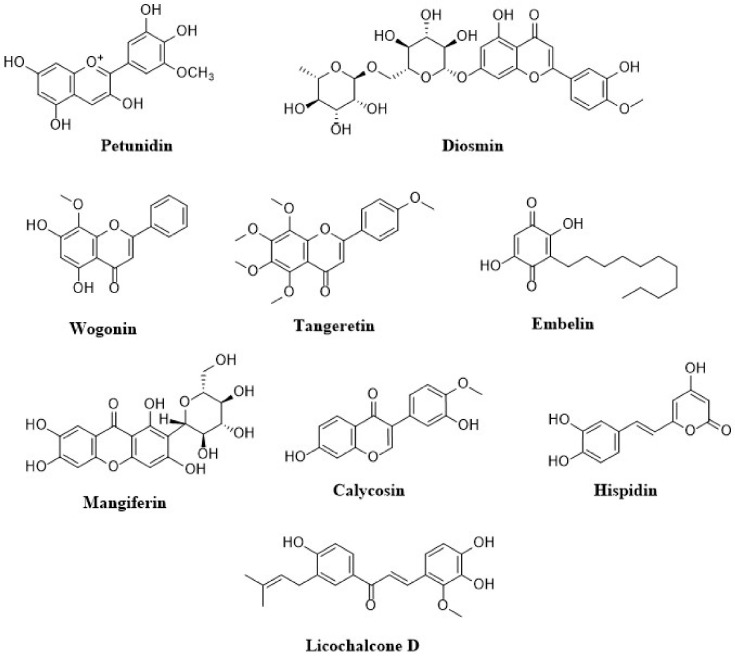
Structures of dietary flavonoids.

Anthocyanins are a flavonoids class that is widely studied. Their notable blue, black, red, and pink colors depend on the pH as well as by the methylation or acylation in the hydroxyl groups on the A and B rings. This characteristic produced high interest in the food industry in a variety of applications. The well-known anthocyanins are cyanidin, delphinidin, malvidin, pelargonidin, and peonidin. Those compounds are present in strawberries, raspberries, blueberries, blackberries, blue corn, black beans, among others (Table 1) [18]. The structures of dietary flavonoids are represented in Figure 2, Figure 3 and Figure 4.

### 2.2. Health Benefits, Medicinal Significance, and Nutraceutical Importance

Flavonoid-rich foods are widely studied and considered as potent bioactive compounds with different biological activities, participating in different important signaling pathways related to chronic disease [23]. Herbal supplements enriched with flavonoids are frequently reported for their ameliorative effects in the management of metabolic syndromes including CVDs and diabetes mellitus. Anthocyanins, like cyanidin and delphinidin 3-glucoside, have shown to improve insulin resistance, insulin production, and hepatic glucose uptake during type 2 diabetes mellitus [24]. Many flavonoids, specifically flavanols, are well known for their antihypertensive effect and endothelial protection by lowering triglycerides and detrimental lipid accumulation. Several flavonoid molecules have been established for their wide range of therapeutic benefits in CVDs including endothelial dysfunction, coronary artery disease, cardiac fibrosis, myocardial infarction, ischemic reperfusion injury, etc. [9,25].

One study suggests that regular consumption of 100 mg of total flavonoids in a day may reduce the risk of developing morbidity as well as fatality due to CVDs by approximately 10% [26]. Due to the presence of multiple hydroxyl groups (-OH) in the flavonoid structure, they exert a strong antioxidant effect and neutralize the oxidative insult during various pathological events [18]. Flavonoids have also been reported as strong inhibitors of DNA damage due to oxidative stress. Nevertheless, flavonoids have also been explored for their positive impact in neurological health and found to be effective on neural regeneration and counter-inflammation in the nerve cells. A study indicated that [6]-epigallocatechingallate, a flavonoid mainly found in green tea, can produce microglial activation and protect against inflammation in Alzheimer’s disease [27]. These days, flavonoids are increasingly being recognized in the field of nutraceuticals for the management of chronic lifestyle-related disorders and the maintenance of healthy aging. Several herbal beverages enriched with a high content of flavonoids are commercially available as anti-aging, antidiabetic and anti-obesity, and blood pressure lowering purposes. For example, hibiscus tea, blue matcha tea, green tea, red tea, rose wine, kiwi wine, and red wine are the most popular beverages commercially available and widely acclaimed for their scientifically proven beneficial health effects.

### 2.3. Antioxidant Potential of Dietary Flavonoids in OS-Induced CVDs

The cardiovascular system is the system most commonly affected by the oxidative stress triggered by spontaneously generated ROS due to the intake of a high-calorie diet, drugs, and other xenobiotics. Mostly, the intake of a high-calorie diet over a long period of time alone can lead to the depletion of myocardial antioxidantstatus and also allows developing chronic abnormalities like endothelial dysfunction, ischemia, and cardiac hypertrophy [28]. Flavonoids consumption has been proven to exhibit a noticeable positive influence in preventing damages produced by ROS and other free radicals in the human body. The beneficial effects of flavonoids have been mostly linked to their strong antioxidant activity. The basic antioxidant mechanism of flavonoids consists in the oxidation of flavonoids by free radicals, resulting in a more stable, less reactive radical [17]. The high reactivity of the hydroxyl group of the flavonoids produces inactivation of the free radicals. Some of the flavonoids can directly scavenge superoxide, whereas other flavonoids can scavenge the highly reactive oxygen-derived radicals like peroxynitrite ions [29]. The preventive action of flavonoids on cardiovascular diseases has been one of the most studied topics. It is well known that the antioxidant activity of these compounds is responsible for the diminution of the oxidative damages of cellular components and induction of cardiomyocytes apoptosis [16,25]. Moreover, another mechanism action of flavonoids is the vasodilation by maintaining the action of the Renin-angiotensin aldosterone system and eNOS in the blood vessel [30]. Flavonoids also have been reported for their anti-apoptotic function on the cardiomyocytes during oxidative insult. Noticeably, fruits and vegetables rich in flavonoids like anthocyanins, and other flavonoids like quercetin, rutin, apigenin, etc., administered to experimental animals exhibited remarkable improvement of the myocardial antioxidant status during drugs (doxorubicin)- and chemical (isoproterenol)-induced cardiac dysfunction [25,27,28].

## 3. Cardioprotective Potential of Dietary Flavonoids

### 3.1. Dietary Flavonoids and Their Health Implications in CVDs

In a metanalysis of prospective cohort studies, regular diets containing flavonoids were accompanied with a lesser risk of CVD mortality. Additionally, consumption of 200 mg/day of total flavonoids is associated with reduced danger of all-cause mortality [31]. Chemically, flavonoids contain a C_6_-C_3_-C_6_ skeleton and consist of 2 aromatic rings (A and B ring). Based on their binding functional group, they are further classified into the subspecies flavonols, flavones, flavanols, flavanones, anthocyanidins, procyanidins, and isoflavones. The hydroxyl radical of flavonoids scavenges free radicals and intercedes antioxidant effects associated with numerous health benefits [17,30]. In the West, the main dietary sources of flavonoids are tea, chocolate, cocoa, vegetables, fruits, red wine, and legumes. In Asian countries such as Japan, soybean is the major source of flavonoids (isoflavones) besides tea, coffee, and legumes [32].

The structural variation in the flavonoid types contributes to their specific activities modulated by their definite molecular pathway. This affects their ADME profile after consumption, thereby altering their bioavailability, target site, and metabolites produced in-vivo. Flavonoids having high absorption are well distributed in multiple tissues while those having limited absorption or distribution exhibit their systemic effects by interaction with microbiota [33]. Colonic microbiota present in our gut can enzymatically break flavonoids into small phenolic acids and aromatic metabolites. These microbiota-generated metabolites curbed the production of cytokines more efficiently when compared with their parent flavonoids. Many of these microbial-derived flavonoid metabolites also provided protection against pancreatic β-cell dysfunction and platelet and monocyte adhesion to the arterial wall [34,35]. Overall, in vitro and in vivo studies suggest that flavonoids exhibit a long range of activities such as antihypertensive effect by inhibiting ACE, potentiating bradykinin effects, decreasing endothelin levels, and increasing NO-mediated vasodilation; anti-apoptotic activity, which lowers the risk of myocardial infarctions; antithrombotic activity; the prevention of LDL oxidation, thereby inhibiting the progression of arteriosclerosis [30,36].

### 3.2. Cardioprotective Mechanisms of Dietary Flavonoids

Over the past decade, a growing interest in scientific research regarding flavonoid consumption to prevent CVDs and to improve vascular health has been noticed. Several studies have shown the advantageous propensities of various classes of flavonoid compounds and flavonoid-enriched plant extracts on the cardiovascular system by balancing the cellular oxidative stress, countering inflammation, and modulating various intracellular signaling pathways [9,24]. Some important molecular mechanisms of the cardiovascular protective function of flavonoids are described below (Table 2).

#### 3.2.1. ROS Scavenging Mechanism

OS plays key role in the development of CVDs including myocardial injury and ischemic heart diseases leading to fatal complications like cardiomyopathy and heart attack, etc. Oxidative insult in the myocardium and endothelial wall occurs due to an imbalance between the generation of ROS/RNS and the clean-up mechanisms of endogenous antioxidant defense systems. Spontaneous generation and accumulation of reactive species (ROS and RNS) accelerates the apoptosis of cardiomyocytes and endothelial cells [84]. Many experimental studies have shown that the antioxidant mechanism of various naturally occurring flavonoids or their active metabolites counters oxidative stress and protects heart tissue during toxic insult [24,85]. However, the ROS scavenging and antioxidant mechanism of individual flavonoids may vary depending on their structural orientation, number and position of hydroxyl groups (-OH), and linkage of the other functional groups to the structural skeleton [30,85].

**Figure 5:**
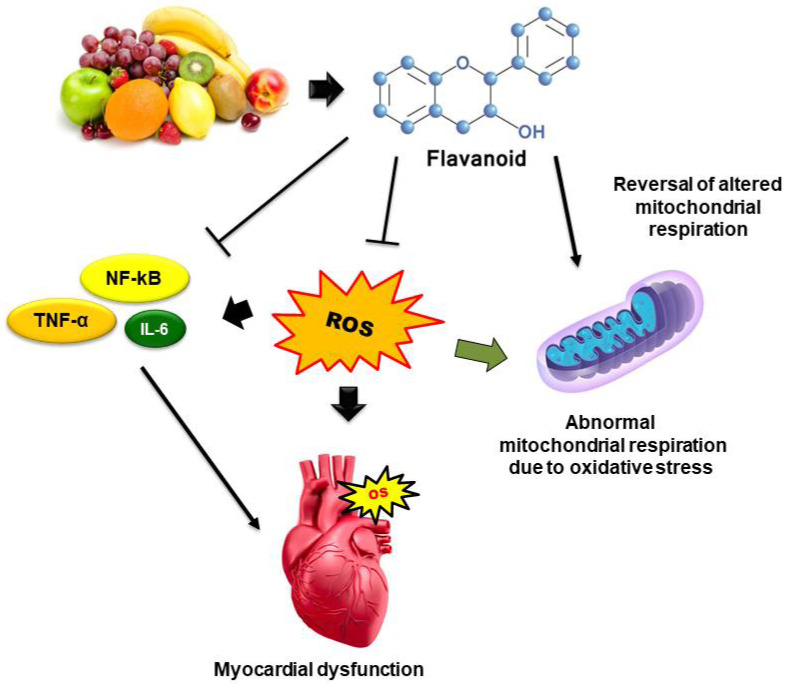
Mechanisms involved in the cardioprotection of dietary flavonoids in OS-associated CVDs. Dietary flavonoids counter myocardial OS via inhibition of endogenous ROS production, down-regulation of inflammatory cytokines (IL-6, TNF-α, NFkB), and reversal of mitochondrial respiratory chain reactions.

Flavonoids may quench ROS by several mechanisms: direct neutralization of the different type (superoxide radical, OH., peroxynitrite radical) of free radicals or ROS; metal chelation property; increase production of endogenous antioxidant enzymes like GSH, SOD, and catalase, etc. and inhibition of cellular ROS-generating enzymes like xanthine oxidase, myeloperoxidase, NADPH oxidase, etc. [30,86]. Various flavonoids which exhibit antioxidant and radical scavenging mechanisms in OS-associated cardiovascular dysfunction are mentioned in Table 2. The basic mechanisms involved in the cardioprotection of dietary flavonoids in OS-associated CVDs are displayed in Figure 5.

#### 3.2.2. Intracellular Antioxidant Signaling Pathways

Unlike the in vitro environment, antioxidative mechanisms of flavonoids in the in vivo system often do not work only on the principle of scavenging free radicals. Rather, flavonoids have been found to activate intracellular antioxidant signaling pathways to accelerate the production of endogenous antioxidants like GSH, SOD, and catalase, etc. [87]. The physiological system comprises various mechanisms to control oxidative stress by accelerating the release of endogenous antioxidants. Nuclear factor erythroid 2, commonly known as Nrf2, is one such important cellular mechanism responsible for the production of endogenous antioxidants during oxidative stress conditions. In normal physiological conditions, Nrf2 couples with KEAP1 protein in the Kelch domain of KEAP1 and spontaneously undergoes degradation in the cytosol [88]. Although mild to moderate oxidative stress triggers dissociation of the Nrf2-KEAP1 complex and translocation of Nrf2 in the nucleus and stimulates upregulation of antioxidant responsive genes like HO1, NQO1, etc., which further accelerates the production and release of endogenous antioxidants like GSH, SOD, and catalase, etc. to control oxidative stress [87,88].

**Figure 6:**
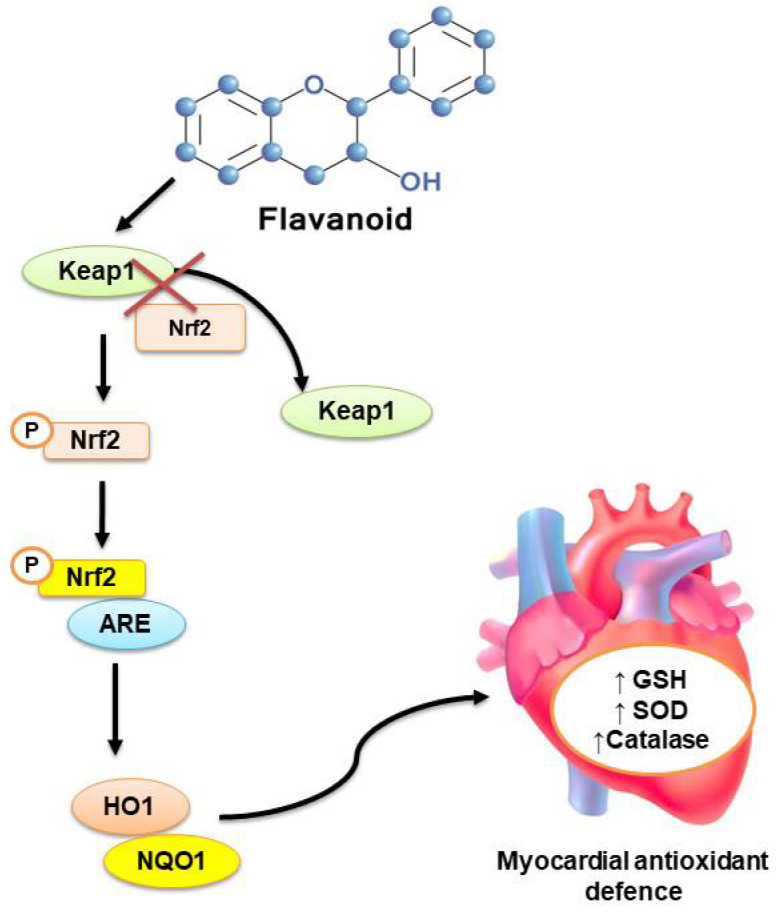
Activation of Nrf2-mediated antioxidant signaling cascade by dietary flavonoids. Nrf2 and Keap1 ubiquitously coupled in the cytosol and lead to the spontaneous destruction of Nrf2. Dietary flavonoids inhibit the Nrf2-Keap1 protein-protein interaction, which results in free Nrf2 to get phosphorylated and bind with the ARE, which activates the downstream antioxidant signaling via up-regulation of HO1 and NQO1.

Flavonoid compounds have been reported to inhibit Nrf2-KEAP1 protein-protein interactions in the cytosol and diminish the spontaneous degradation of Nrf2 protein. Flavonoids competitively bind with the Keap1 protein in the Nrf2 binding site resulting in the translocation of Nrf2 protein into the nucleus and activating the downstream proteins HO1 and NQO1 [88]. Activation of these downstream proteins directly influences the up-regulation of antioxidant genes like GSH, SOD, and catalase (Figure 6). For example, flavonoids like quercetin, luteolin, baicalin, genistein, wogonin, etc. have been found to protect the heart via activation of the Nrf2 pathway during chemical-induced myocardial infarction and cardiotoxicity [88,89].

#### 3.2.3. Counter-Inflammatory Pathways

Inflammation is thought to be one of the most aggravating factors in the progression of a variety of CVDs, from endothelial dysfunction to myocardial apoptosis [90]. Inflammation occurs due to the increased oxidative stress and elevated level of ROS in response to injurious stimuli and in conjunction with the multiple complex signaling pathways. A short-term inflammation is the result of immunological response to the body; however, chronic inflammation in the cardiovascular system leads to the development of pathological incidents in myocardial tissue and blood vessels. During chronic inflammation, pro-inflammatory cytokines such as IL-1, IL-6, and TNF- cause damage to the myocardial and vascular tissue, resulting in myocardial infarction and hypoxia in cardiomyocytes, which leads to apoptosis. Similarly, increased inflammation substantially damages the endothelial wall resulting in the development of a ischemic condition [85,90]. Oral flavonoids supplementation is extensively reported to produce decreased inflammatory cell invasion, lowered levels of pro-inflammatory cytokines and tissue fibrosis, and increased cell survival and function, according to epidemiological studies. Inhibition of signaling through NF-kB (nuclear factor-B) seemed to be a central pathway that seemed to mediate the anti-inflammatory effect of several flavonoids [85,91]. Many flavonoids, in general, can exert cardioprotective effects by modulating multiple targets and genes involved in major pathways such as MAPK/ERK/JNK/p38 impairment, modulation of PI3K-Akt-eNOS, the STAT3 pathway, and the AMPK-mTOR pathway [30,85]. Other anti-inflammatory mechanisms of flavonoids involved during cardiovascular oxidative stress are up-regulation of SIRT1, SIRT3, VEGF-B, pAkt, GSK3, and Bcl-2 genes and down-regulation of TLR-4, COX-1,COX-2, FAK, ET-1, Caspase 9, and Bax genes [92].

#### 3.2.4. Mitochondrial and Intracellular Pathways

Mitochondria play a vital role in the normal functioning of cardiomyocytes and endothelial cells. Synthesis of ATP by catabolism of carbon-rich sources via oxidative phosphorylation is one of the major roles of mitochondria. The integrity of the inner mitochondrial membrane is very much essential to normal physiological and biophysical functioning [93]. Mitochondrial damage during oxidative insult like the accumulation of cardiotoxins or due to ischemia/reperfusion is considered a key event leading to cardiomyocytes dysfunction and apoptosis [94]. In this regard, the protective potential of various flavonoids on mitochondrial functions has been widely investigated. The mechanism of action of certain flavonoids on mitochondrial targets may be another reason for the cardioprotective effect, which is enabled by maintaining mitochondrial ATP output and calcium homeostasis, as well as preserving mPTP opening and subsequent cell apoptosis [94,95]. Many flavonoid compounds—for example, epigallocatechin3-gallate, baicalein, puerarin, naringenin, etc.—have been reported to exhibit cardioprotection during oxidative stress via activation of mitochondrial ion channels present in the inner mitochondrial membrane-like mitoK, mitoKATP channels [96,97]. Another study suggested that dietary flavonoid consumption also acts as a cardioprotective agent by activation of Ca^+2^ channels and modulation of mitochondrial Ca^2+^ uptake [94].

Oxidative phosphorylation and maintenance of respiratory chain or electron transport chain are the vital functions of mitochondria. However, oxidative insult in the cardiac tissue hampers the complex formation (Complex I) and subsequently releases cytochrome C [94,96]. Notably, anthocyanin flavonoids like cyanidin 3-O-glucoside and delphidin 3-O-glucoside have been found to reduce oxidative stress in cardiac cells by restoration of mitochondrial bioenergetics and safeguarding the preservation of normal functioning of the complex I [98]. Flavonoids have also been found to suppress the generated ROS due to mitochondrial respiration by directly inhibiting enzymes and chelating the trace elements involved in ROS generation [94]. Evidently, flavonoids prototypes like quercetin, kaempferol, and epicatechin, etc. have been found to inhibit H_2_O_2_ production in isolated rat heart mitochondria [99].

## 4. Pharmacokinetic and Toxicological Issues

### 4.1. Bioavailability and Biotransformations of Dietary Flavonoids

Although flavonoids have shown countless health benefits, their low oral bioavailability has been a major concern in drug development. Absorption and distribution of flavonoids and their metabolites from the gut to the blood stream are the important phenomena to achieve the optimum therapeutic efficacy. Also, to understand the bioactivity and mechanism of action of dietary flavonoids in the body, it is fundamental to determine how much and which chemical forms they reach in systemic circulation, as these would be the physiologically active forms [100]. The most important factors which are associated with the absorption and bioavailability of dietary flavonoids are their types, number and position of sugar linkage, metabolism via phase II metabolic enzymes, and gut microbiota [101]. In foods, flavonoids are often present in their glycosylated form; but once they are ingested, the sugar moiety is removed before the absorption phase. This mechanism is carried out in the brush border of the small intestine by the enzyme lactase phlorizin hydrolase (LPG) that produces the hydrolyzation of the structure and the sugar is removed to release the aglycone to enter in the epithelial cells by passive diffusion. Organic anion transporter (OAT) families SLC22A, SLC21A, and MRP are also responsible for the absorption and delivery of flavonoids around the body as well as their excretion in urine [102].

The food matrix and where flavonoids exist in the dietary sources play an important role in the absorption and bioavailability of various flavonoids. Evidently, ethanol present in red wine enhances the absorption of anthocyanins from the gut [102]. Flavonoid (for example, quercetin) co-administration with carbohydrate-containing foods exhibited enhanced absorption in the intestine and bioavailability. A fatty matrix can increase the uptake of flavonoids and slow down their clearance. On the other hand, protein co-administration and flavonoid protein interactions significantly reduce the oral bioavailability of many flavonoids [103].

The aglycones of flavonoid glycosides undergo metabolic conversion or modification before passing into the blood stream, presenting sulfate, glucuronide conjugate, and/or methylated metabolites through the action of sulfotransferases, uridine-5′-diphosphate glucuronosyltransferases (UGT), catechol-O-methyltransferases (COMT), and glutathione transferees [104]. When metabolites reach the bloodstream, they are subjected to phase II metabolism with transformations taking place in the liver, prior to urinary excretion. Cytochrome P450 (CYP450) superfamily in the liver microsomal enzymes mostly bear the responsibilities of phase II metabolism. Mostly CYP1A2 and CYP3A4 are demonstrated to be the key enzymes in human liver mediating the oxidative de-methylation of many flavonoid compounds in the A and B ring [105].

**Figure 7:**
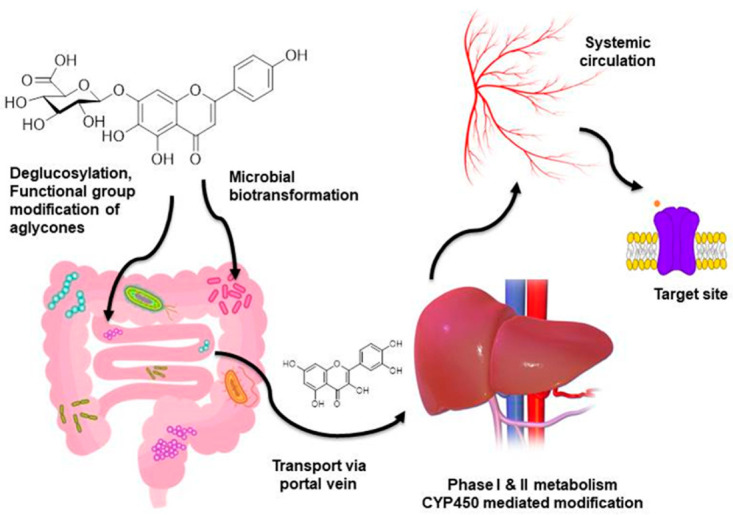
Schematic of bioavailability and metabolism of dietary flavonoids. Flavonoids from dietary sources after ingestion go through de-glycosylation and modifications like sulphate conjugation, glucuronide conjugation, etc. in the small intestine and enter the liver via the portal vein. Hepatic microsomal enzymes (CYP450 isoforms) take major responsibility to convert the flavonoid aglycones into the simpler form. After hepatic first-pass metabolism, metabolites reach the systemic circulation and finally bind to the target site. Colonic gut microbiota also plays a similar role in the de-glycosylation and biotransformation through microbial enzymes.

Another important mechanism of non-absorbed flavonoids in the small intestine consists in the passing of flavonoids into the distal colon where the intestinal microbiota makes some changes and produces phenolic acids and aromatic compounds that can enter in the phase II metabolism and are excreted in the urine [106]. Recently, it has been proven that the gut microbiota plays a significant role in the metabolic conversion of many flavonoids as well as other phenolic compounds present in the dietary sources. Beneficial micro-organisms like lactobacillus in the gut release enzymes like phenolase, glucosidase, etc., which eventually transform the parent compounds into several newer metabolites with high bioavailability [107]. Biotransformation not only caters to the clearance of the flavonoids from the human body but also facilitates the molecular interactions with the therapeutic target. It is also proven that the therapeutic properties exerted by the many naturally occurring flavonoids and phenolics are because of their metabolites but not the actual compounds due to their several biopharmaceutical limitations. A schematic of bioavailability and metabolism/biotransformation reactions of dietary flavonoids is depicted in Figure 7.

### 4.2. Toxicities and Interactions with Drugs/Foods/Herbs

In contrast to the beneficial effects of flavonoids, the toxic effects and interactions with drugs/foods/herbs and other phytochemicals have been less explored. Nevertheless, scientific interest to uncover the toxicity profile and chemical/physicochemical/biological interactions of flavonoids and their possible metabolites is continuously increasing. A wide variety of flavonoid compounds have exhibited cytotoxic effects to various cancer cells and inhibit tumor progression substantially by acting as pro-oxidants and inducing mitochondrial oxidative stress and also leading to DNA damage [108]. Many vegetables, fruits, and medicinal herbs enriched with flavonoids are also found to exhibit anti-proliferative properties against cancer cells. On the contrary, flavonoids and flavonoid-enriched foods/herbal extracts often demonstrated no or mild cytotoxicity in normal cells only with a very high concentration. A possible explanation for these conflicting phenomena is that they may be due to the selective toxicity of flavonoids to cancer cells and differences in their cellular physiology and biochemical events than the normal cells [109].

The interest in using flavonoids as food supplements and/or nutraceuticals alone or together with other prescription medicines are increasing, which may lead to a risk of flavonoid-drug/herb/food interactions. According to certain published reports, some dietary flavonoids may have the potential to interact adversely with clinically used drugs. Dietary flavonoids alone or a combination present in dietary sources were often found to alter the pharmacokinetic profile of therapeutic drugs [109,110]. Many herbal drugs enriched with flavonoids have been reported to accelerate or diminish the rate of absorption of various drugs when co-administered. One of the most studied mechanisms of dietary flavonoids leading to increased or decreased bioavailability of the therapeutic drug is CYP450 enzyme interaction. Dietary flavonoid compounds individually or present in dietary supplements or herbal preparations were found to inhibit or induce various isoforms of CYP450 enzyme in the gut and liver and also found to modify the action of xenobiotic efflux in the gut [111,112]. This phenomenon was often found to increase the bioavailability of many drugs, which is of course beneficial for the drugs with low bioavailability or metabolic stability. However, these pharmacokinetic alterations turn negatively for drugs with an extremely narrow therapeutic index like digoxin, lisinopril, captopril, etc. [111]. The interactive behavior of dietary flavonoids and alterations of pharmacokinetics are not always predictable. One of the main reasons behind this effect is that the concentrations of individual flavonoids and other non-flavonoid constituents are different in every matrix. Toxicity on the other hand is a dose and concentration-dependent phenomena. Consumption of dietary flavonoids as food or supplements generally produces low concentrations of flavonoids during daily dietary intake. On the other hand, high doses of flavonoids in food supplements can become pro-oxidants and generate free radicals rather than acting as antioxidants [110]. Hence, it is very important to have a better understanding of the timing and amount of intake of dietary flavonoids in order to maximize the benefits while minimizing the risks. Some important flavonoid-drug interactions are depicted in Table 3.

### 4.3. Strategies to Overcome Pharmacokinetic and Toxicological Limitations

The delivery of phytochemicals like flavonoids is challenging due to poor solubility, run-down permeability, low bioavailability, instability in the biological environment, and extensive first-pass metabolism. Recently, various absorption-enhancing techniques have been developed and used to improve the oral bioavailability and efficacy of poorly absorbable flavonoids by increasing their solubility or gastrointestinal permeability and preventing metabolic degradation. Researchers across the globe have proposed several approaches including structural modifications of the parent compound, nano-formulation, matrix complex formation, co-crystal technique, and dispersion techniques, etc. to enhance the pharmacokinetics and bioavailability of natural active flavonoids and improve their efficacy [113]. Colloidal drug delivery systems (CDDS) as carriers for phytochemicals have seen an exponential rise and have also helped in the rejuvenation of ancient and forgotten natural molecules by optimizing some unfavorable chemical or physical properties of the natural active compounds, including solubility and the biological stability, while, on the other hand, also improving their radical scavenging activity and promoting bioavailability [114]. The delivery system is capable of increasing the antioxidant activity of flavonoids by preventing degradation of the formulation due to encapsulation and maintaining the drug concentration over time, which in turn increases the antioxidant/radical scavenging activity of the active compound compared to the unloaded one. Furthermore, these also help in compounding sustained and controlled release formulations which can be used for flavonoid-targeted therapies [115]. In comparison to the conventional formulation, micro or nano-emulsion increases the penetration rate through biological membranes and also enhances their ADME phase, thereby decreasing associated toxicities [116]. The use of biopolymers in formulations used for CVDs treatment adds an advantage because of its favorable properties such as biodegradability, good biocompatibility, and attractive biomimetic characteristics [117]. Structural modification of the parent flavonoid compounds also has been proven as one of the successful strategies to overcome poor solubility and GI absorption. Glycosylation and glucuronide conjugation are the useful tailoring reactions which may significantly change the physicochemical properties of hydrophobic flavonoids. The introduction of new polar groups or masking the selective functional groups in the structural skeleton, which is popularly known as the pro-drug approach, have become useful to improve the pharmacokinetic profile of various dietary flavonoids [118]. It is often observed that co-administration of food and flavonoids together produces better absorption of flavonoids from the gut. Hence, the complex carrier formation approaches like cyclodextrin complex or lipid/carbohydrate-flavonoid conjugate are some of the approaches to overcome pharmacokinetic limitations [104,112]. The formulation of nanoparticles or nanocrystals is the most common approach to enhance the absorption and bioavailability of flavonoids and has been found to be remarkably effective in cancer chemoprevention [119,120]. However, all these strategies to improve the pharmacokinetic profile of dietary flavonoids are exclusively dependent on the area of their application and most of them are still under experimental investigational phases and need more in-depth studies to make any conclusive statement.

## 5. Therapeutic Approaches and Future Drug Discovery

Flavonoids are allied with a wide spectrum of health-promoting effects and therefore are a requisite component in a variety of nutraceutical, medicinal, and cosmetic applications. These compounds exhibit a wide variety of medicinal properties such as anti-mutagenic, anti-atherosclerotic, cardiovascular protective, antidiabetic, insulin sensitizer, anti-carcinogenic, antioxidant, anti-inflammatory, antithrombogenic, and antitumor agents [16,17]. Flavonoid supplementation exhibited positive improvements during neurodegenerative complications like Alzheimer’s disease [27]. In anticancer therapy, flavonoids have been extensively used. Flavonoids were used as a single agent or in combination with other therapeutics against hematopoietic/lymphoid or solid cancers in 22 phase II and 1 phase III clinical trials (PubMed, Scopus, and Web of Science) released by January 2019. Quercetin is one of the most studied flavonoids in the mitigation of cancer and related complications [121]. Flavonoids have also been known for their antimicrobial activity and many of them have been isolated and identified as having properties of antifungal, antiviral, and antibacterial activity. Many flavonoid molecules have been used in combination with synthetic and other existing antibiotics to increase the efficacy and overcome drug resistance [122]. Naturally occurring flavonoid scaffolds often offer novel templates to design various potent synthetic drugable molecules. For example, phlorizin is a chalcone type of flavonoid which brings the idea of clinically approved SGLT-2 inhibitor gliflozins [123]. The most intriguing properties of flavonoids in the field of disease management are their antioxidant and cytoprotective properties during oxidative stress. Because of this property, flavonoids hold an irreplaceable position in the fields of nutrition, food safety, and health. Various flavonoid-enriched nutraceuticals like green tea, matcha tea, and beverages are gaining global interest [124]. Flavonoids such as quercetin, naringin, hesperetin, and catechin possess a higher grade of antiviral activity and they act by affecting the replication and infectivity of certain RNA and DNA viruses [125]. Recently, during this COVID-19 pandemic, there is an overwhelming scientific interest in searching for naturally occurring and synthetic flavonoid compounds to reduce COVID-19-infected cardiovascular malfunctioning by blocking the viral entry at the ACE2 receptor [126].

Despite their broad and multi-potent pharmacological properties, research into the therapeutic efficacy of standardized flavonoid products extracted from plant sources in prospective human studies is still missing. To produce cost-effective flavonoid-based natural health products, scale-up, consumer- and environment-friendly green technologies are needed. Flavonoid supplementation should be performed with caution in cancer patients because it can interfere with radiotherapy and various chemotherapies. There should be a strict monitoring of the flavonoid-rich food-drug interactions as well to minimize the unwanted contraindications. To resolve bioavailability issues, improve targeted delivery, and improve the therapeutic efficacy of certain flavonoids, multidisciplinary research collaborations are needed. The biotransformation of flavonoids is also a major concern in its drug development aspects. Microsomal- and gut microbiota-mediated metabolism of a large variety of dietary flavonoids is still not well studied, which can give ideas on how to design novel and therapeutically active potent small molecules and also open up newer directions for therapeutic strategies.

## 6. Conclusions

Dietary flavonoids are bioactive components of fruits and vegetables that may be effective in the prevention of diseases such as cancer and CVDs. Current research trends on flavonoids aim to identify plant-derived/dietary flavonoids with regard to exploring their medicinal applications and/or biological/pharmacological activities in various chronic disorders. The bioactivity of flavonoids depends on their pharmacokinetic, metabolic, and pharmacodynamic profile in the human body. Information embedded in this review would help researchers to understand the biochemical (molecular) mechanisms of action, bioavailability, metabolism and other pharmacokinetic aspects, and toxicities/safety concerns of dietary flavonoids possessing beneficial health effects in various CVDs.

## Figures and Tables

**Table 1:** Dietary flavonoids with their natural sources and health benefits [18,21,22].

Flavonoids	Major Flavonoids	Major Source	Health Benefits
Flavonols	Isorhamnetin Kaempferol Myricetin Quercetin	Onions, broccoli, tea, apple, blueberries	Regulates systolic blood pressure, glycemic levels, and BMI.
Flavones	Apigenin Luteolin	Parsley, celery, chamomile tea, fenugreek, onion, garlic, pepper, citrus fruits	Regulates blood glucose levels.
Flavanones	Eriodictyol Hesperetin Naringenin	Citrus fruits, mint, tomatoes.	Lowers risk of ischaemic stroke.
Flavanols	Catechins Epicatechins	Apricots, cocoa, chocolates, red grapes, red wine, tea	Reduces mean arterial pressure. Improves insulin resistance and LDL-C, HDL-C levels.
Procyanidins	Theaflavins Thearubigins	Cocoa, apples, grapes, red wine, chocolates	Regulates blood pressure.
Anthocyanidins	Cyanidin Delphinidin Malvidin Pelargonidin Peonidin Petunidin	Berries, red wine, red cabbage, bright colored fruits, cherries, cranberries	Lowers risk of Myocardial infarctions.
Isoflavones	Daidzein Genistein Glycitein	Soyabean, dairy products, egg, meat	Beneficial for T2DM.

**Table 2:** Cardioprotective effects of dietary flavonoids in OS-induced CVDs.

Flavonoids	Oxidative Stress Model	Molecular Mechanism	Reference (s)
Apigenin	Myocardial ischemia-reperfusion injury in h9C2 cardiomyocytes; adriamycin-induced cardiotoxicity in Kunming mice	↑PI3K/AKT/mTOR pathway	[37,38]
Apigenin-7-O-b-D-(-6′′-p-coumaroyl)-glucopyranoside	Primary neonatal cardiomyocyte (C57/6J) ischemic reperfusion model in vitro	↑PKCe translocation signaling ↑Nrf2/HO-1 pathway ↓NF-kB signaling Pathway	[39]
Dihydromyricetin	Doxorubicin-induced cardiotoxicity	↑SIRT1 ↓NLRP3 inflammosome	[40]
Quercetin	Isoproterenol-induced cardiac fibrosis	↑Nrf2-HO; ↓LDL receptor expression; ROS scavenger	[41]
Icarin	High glucose- and adenovirus-induced cardiomyopathy in neonatal C57 mice	↑Apelin/SIRT3	[42]
Isoliquiritigenin	Hypoxia-induced contractile dysfunction in cardiomyocytes	↑AMPK and ERK signaling pathways; ROS scavenger	[43]
Scutellarin	Isoproterenol-induced myocardial infarction in SD rats	↓α-SMA ↑CD31, Jagged1, Notch 1, and Hes1	[44]
Cyanidin-3-glucoside	Wistar rats induced by STZ	↑TIMP-1 ↓MMP-9,.TGF-b, p-MEK1/2, CTGF, P-ERK1/2, FGF2	[45]
Morin	Isoproterenol-induced myocardial infarction; doxorubicin-induced cardiac fibrosis	Restored the mitochondrial function and improvement of mitochondrial antioxidant enzymes; ↓myocardial; Apoptosis; ↑Bcl-2	[46,47]
Fisetin	Isoproterenol-induced cardiac ischemia	↓RAGE and NF-κB; ↓Bax, caspase-3, cytochrome-c; ↑Bcl-2; ↓Myocardial apoptosis	[48]
Rutin	Cobalt chloride-induced hypoxic injury in H9c2 cells	Modulation of Akt, p-Akt, p38 and p-p38; ↓of HIF-1α, BAX and caspase	[49]
Acacetin	Doxorubicin-induced cardiomyopathy	↑Sirt1/pAMPK pathway ↑AMPK/Nrf2 signal pathway	[50]
Hesperidin	Nitric oxide deficiency-induced cardiovascular remodeling	↓TNF-R1 and TGF- β1 protein expression; ↓MMP-2 and MMP-9	[51]
Luteolin	Doxorubicin-induced cardiotoxicity	↑AKT/Bcl-2 signaling pathway; ↑Nrf2/HO-1 pathway; ↑eNOS/Nrf2 signaling pathway	[52,53]
Baicalein	t-BHP-induced oxidative stress; H_2_O_2_ and ischemia/reperfusion (I/R) stress	↑Nrf2/Keap1 pathway; ↓KLF4-MARCH5-Drp1 pathway	[54,55]
Baicalin	Hypoxia-induced oxidative stress in cardiomyocytes; Angiotensin-II-induced endothelial dysfunction	↑Nrf2/HO-1 signaling pathway; ↓NF-kB signaling pathway; ↓iNOS protein expression	[56,57]
Astragallin	Myocardial ischemia/reperfusion (I/R) injury in isolated rat heart	↓ROS; ↓ Inflammation; ↓Myocardial apoptosis; ↑Bcl-2	[58]
Cyanidin-3-O-glucoside	Myocardial ischemia-reperfusion injury in SD rats and H9c2 cells	↓USP19, Beclin1, NCOA4, and LC3II/LC3I; ↓LC3II/LC3I; ↓TfR1 expression; ↑FTH1 and GPX4; ↓Ferroptosis promoter RSL3	[59]
Hyperoside	High glucose-induced oxidative stress in cardiac cells	↑ p-AKT/AKT and p-Nrf2/Nrf2; ↓Myocardial apoptosis and levels of ROS and MDA	[60]
Chrysoeriol	Doxorubicin-induced toxicity in cardiomyocytes	↓ROS, MDA; ↑GSH, SOD	[61]
Orientin	Myocardial ischemia reperfusion injury	↑AMPK, Akt and Bcl-2; ↓mTOR and Raptor, Beclin 1	[62]
Vitexin	Myocardial ischemia/reperfusion (I/R) injury	↓phospho-c-Jun; ↑phospho-ERK; ↓inflammatory cytokines and ↓MAPK pathway.	[63]
Kaempferol	Cardiac hypertrophy by aorta banding	↓ASK1/JNK1/2/p38 signaling pathway; ↓ASK1/MAPK signaling pathways (JNK1/2 and p38)	[64]
Naringin	High-cholesterol-diet-induced endothelial dysfunction and oxidative stress in rats	↓LOX-1, NADPH oxidase subunits (p47phox, Nox2, and Nox4), and iNOS	[65]
Naringenin	H_2_O_2_-induced oxidative stress in cardiomyocytes	↓ROS; ↑Nrf2 signaling pathway	[66]
Tilianin	Myocardial ischemia/reperfusion injury in rats	↑AMPK, pAMPK, SIRT1, PGC-1alpha, NRF1, TFAM and FOXO1 proteins	[67]
Spinosin	Myocardial ischemia/reperfusion injury in rats	↓GSK3β; ↑PGC-1α; ↑Nrf2/HO-1 pathway	[68]
Myricetin	Myocardial ischemia/reperfusion injury in rats	↓STAT1	[69]
Delphinidin	Myocardial ischemia/reperfusion injury in rats	↓STAT1	[69]
Daidzein	Isoproterenol-induced apoptosis in H9c2 cardiomyoblast	↑Akt activation	[70]
Genistein	Doxorubicin-induced cardiotoxicity	↑Nrf2/HO-1 signaling pathway; ↓DNA damage	[71]
Malvidin	Isoproterenol-induced apoptosis in H9c2 cardiomyoblast	↑Nrf2/HO-1 signaling pathway; ↓NF-κB signaling pathway activation	[72]
Petunidin	Myocardial ischemia/reperfusion injury in rats	↑Bcl-2 protein expression, ↓ NOX4 and Bax expression, ↓cytoplasmic cytochrome c expression; ↓ROS	[73]
Aspalathin	Doxorubicin-induced cardiotoxicity in cardiomyocytes	↓ROS; ↓ Myocardial apoptosis	[74]
Diosmin	Myocardial ischemia/reperfusion injury in rats	↑Bcl-2 expression; ↑antioxidant enzyme activities; ↓LPO	[75]
Wogonin	Isoproterenol-induced myocardial infarction	↑Nrf2/HO-1 signaling pathway; ↓Inflammation	[76]
Tangeretin	Isoproterenol-induced myocardial infarction	↑PI3K/Akt signaling pathway	[77]
Embelin	Isoproterenol-induced myocardial injury	↑Bcl-2; ↓Bax, Cytochrome c, cleaved-caspase-3 & 9 and PARP;	[78]
Neferin	Isoproterenol-induced myocardial injury	↓Inflammation; ↑ Tissue antioxidant status	[79]
Mangiferin	Myocardial ischemia/reperfusion injury in rats	↓Phosphorylation of p38 and JNK, phosphorylation of ERK1/2; ↓TGF-β, ↓MAPK	[80]
Calycosin	H_2_O_2_-induced oxidative stress in cardiomyocytes	↓ Apoptosis; ↑ ER/ and Akt	[81]
Licochalcone D	Myocardial ischemia/reperfusion (I/R) injury in cardiomyocytes	↓ Caspase 3 and PARP; ↓ IL-6, NF-kB and p38 MAPK	[82]
Hispidin	H_2_O_2_-induced oxidative stress in cardiomyocytes	↓ Apoptosis, ROS, DNA damage, caspase 3 and Bax expression ↑ HO-1, CAT, Bcl-2, Akt/GSK3 and ERK ½	[83]

**Table 3:** Flavonoid-drug interaction [111].

Drugs	Flavonoid	Species in Which Tested	Change in Bioavailability
Diltiazem (15 mg/kg, oral)	Morin (1.5–7.5 mg/kg, oral)	Rat	1.4- to 1.8-fold increases
Talinolol (10 mg/kg, oral)	Naringin (1–20 mg/kg, oral)	Rat	1.5- to 3.0-foldincreases
Etoposide (6 mg/kg, oral)	Morin (15 mg/kg, oral)	Rat	1.4-fold increases
Digoxin (0.02 mg/kg, oral)	Quercetin (40 mg/kg, oral)	Pig	1.7-foldincreases
Moxidectin (0.2 mg/kg, subcutaneous)	Quercetin (10 mg/kg, subcutaneous)	Sheep	1.8-fold increases
Verapamil (10 mg/kg, oral)	Quercetin (15 mg/kg, oral)	Rabbit	2-fold increases
Paclitaxel (30 mg/kg oral)	Genistein (10 mg/kg, oral)	Rat	1.5-fold increases

## Data Availability

Not applicable.
